# Supplementary material for: Complexome profiling of the Chlamydomonas psb28 mutant reveals TEF5 as an early PSII assembly factor
Source: Plant Cell. 2025 Mar 17;37(6):koaf055. doi: 10.1093/plcell/koaf055 (PMC12164590; doi:10.1093/plcell/koaf055)
Supplement: koaf055_Supplementary_Data [file koaf055_supplementary_data.zip › psb28_interactive.zip!/plots/intensity/qualityPlots/peptides/_Cre12.g499800.t1.2.html]

### Cre12.g499800.t1.2

Trivial name:   
SeqCoverage [median]: 51.60  
Mass: 10.48 kDa  
  
 

| Sequence | MissedCleavage | Mass [Da] | wtIntensity | muIntensity | Score |
| --- | --- | --- | --- | --- | --- |
| EVTMAEYNATPLVYLQSPDR | 0 | 2296.10 | 706.03 | 0.00 | 42.17 |
| EVTMAEYNATPLVYLQSPDRHPTR | 1 | 2787.36 | 22102.67 | 119645.60 | 181.61 |
| SYSAYFTR | 0 | 993.46 | 37298.10 | 77167.12 | 170.66 |
| VPGMSDVPHAYDELMHK | 0 | 1924.88 | 14454.85 | 23392.54 | 181.58 |
|  |  |  | 74561.7 | 220205.3 |  |
